# Multi-level change strategies for health: learning from people-centered advocacy in Uganda

**DOI:** 10.1186/s12939-022-01717-1

**Published:** 2022-09-29

**Authors:** Angela Bailey, Vincent Mujune

**Affiliations:** 1 Accountability Research Center, American University School of International Service, Washington, D.C., USA; 2 StrongMinds in Uganda, Kampala, Uganda

**Keywords:** Health accountability, Health advocacy, Social accountability, People-centered advocacy, Multi-level advocacy strategies, Participatory governance, Process monitoring, Community monitoring

## Abstract

**Background:**

The paper analyzes how the Accountability Can Transform Health (ACT Health) program activated bottom-up citizen action to secure government responses and more accountable health services in Uganda. The ACT Health program had two phases—Phase 1 focused on a community-level intervention studied with a randomized control trial, and Phase 2 supported citizen-led advocacy targeting government officials across multiple levels. The focus of this paper is an analysis of Phase 2, when the “people-centered advocacy” approach supported almost 400 community advocates representing 98 health facilities to organize, identify joint advocacy priorities, directly monitor health services, and collaborate on health advocacy campaigns in 18 districts. Most district campaigns focused on the complex, power-laden issue of health worker absenteeism. With a few notable exceptions, iterative cycles of engagement between citizens and the state across multiple levels are infrequently discussed in the formal literature on health accountability.

**Methods:**

This paper is based on a comparative, inductive, practitioner-led analysis of program monitoring data from 18 multi-level health advocacy campaigns. The findings emerge from analysis of a “Heat Map,” capturing grounded accounts of government responses to community-led advocacy.

**Results:**

Officials in eight out of 18 districts fulfilled or surpassed commitments made to community advocates. Government responses included: increased monitoring, more downward accountability, countering backlash against advocates, applying sanctions for absent health workers, and increased budget allocations. Advocates’ bottom-up advocacy worked in part through triggering top-down responses and activating governmental checks and balances.

**Conclusions:**

Methodologically, this article demonstrates the value of analyzing process monitoring and program data to understand outcomes from direct engagement between citizens and the state to improve health services. Survey-based research methods and quantitative analysis may fail to capture signs of government responsiveness and relational outcomes (such as subtle signs of shifting power dynamics) many hope to see from citizen-led accountability efforts. Practitioners’ perspectives on how accountability for health emerges in practice are important correctives to much positivist research on accountability, which has a tendency to ignore the complex dynamics and processes of building citizen power.

## Background

Accountability work is highly relational, involving citizens, civil society actors, and complex governing institutions at multiple levels of the health system. Many studies of accountability work focus primarily on one level of engagement: that between citizens and frontline health service providers—often the least powerful actors in complex health systems. This paper offers practitioner perspectives and analysis of how the Accountability Can Transform Health (ACT Health) program activated bottom-up citizen advocacy campaigns to directly engage officials and powerholders across multiple levels of the governance system to secure more responsive and accountable health services in Uganda.

Providing an account of how citizen-led advocacy triggered a range of responses from government power-holders across multiple levels of the health system, this paper summarizes findings from a longer comparative analysis of 18 multi-level health advocacy campaigns [1]. Civil society organizations (CSOs) supported community advocates to directly engage district and national-level officials on priority concerns in their communities. Almost 400 community advocates representing 98 health facilities in 18 intervention districts organized and collaborated on health advocacy campaigns with many focusing on the complex problem of health worker late-coming, late departure, and absenteeism relative to government standards (referred to collectively as "absenteeism" in this paper). This “people-centered” advocacy approach placed citizens in agenda-setting and action roles. Community advocates drove campaigns targeting village, parish, subcounty, and district officials. With accompaniment and strategic support from CSOs, advocates engaged national-level politicians and officials, bringing the community concerns on absenteeism directly into national campaigning on priority health concerns.

Practitioners’ perspectives on how accountability for health emerges in practice are important correctives to much positivist research on accountability, which tends to ignore the complex and messy processes of building citizen power. Experimental and quasi experimental intervention studies of accountability are seen as ‘rigorous’ but it is acknowledged that they often focus on short causal chains and narrowly measurable inputs and outcomes. Experimental research—particularly randomized control trials—typically test simple interventions, often focused on engagement between citizens and frontline health service providers. This has meant ignoring the numerous, less linear, and more complex processes through which citizens are able to grow their capacities to demand better services, and to enforce those demands. Even the best external researchers may miss some of the subtleties and nuances to understanding and interpreting the data from a complex intervention. Based on a systematic review of program process monitoring data, this paper focuses on sharing the practitioners’ perspectives as a critical counterpoint to the analytical limitations of experimental research.

The Background section provides a brief overview of the ACT Health program in the Uganda context. The Approach and Methods section briefly describes the data sources and review processes used for this inductive, practitioner-led analysis. The Findings section catalogs observed outcomes—the range of government responses to community-led advocacy campaigns and the outcomes for participating community advocates themselves. The Discussion section offers practice-based insights on the possible mechanisms at work and the meaning of the observed outcomes in the Uganda context. The Conclusion offers some final reflections on the implementation and study of strategic accountability work.

Uganda’s legal and policy provisions enable citizen participation in planning and monitoring government services in theory, but these spaces are often inaccessible in practice. Centralized political power, proliferation of subnational government entities, complex health governance, and narrowing civic space curtail the abilities of civil society and citizens to directly engage government powerholders. Even when mandated for official government processes, “community participation” is frequently passive, with affected people consulted about pre-determined agendas, but rarely supported to advocate directly. This is rooted in Uganda’s political culture and history, which “dictated obedience and deference towards people in positions of power and authority” and early opportunities for citizen participation did not erase memories of victimization by people in positions of power [2]. CSO-led accountability work is prolific in Uganda, yet organizations and funders often underestimate the difficulties in chipping away at the “invisible power” that shapes the psychological and ideological boundaries of participation [3–5].

Between 2012 and 2018, a small consortium of civil society organizations (see acknowledgements) designed and implemented the Accountability Can Transform Health (ACT Health) program, to foster direct engagement between citizens and public servants from frontline health workers up to national-level powerholders. The overall goal was to improve accountability for quality service provision and encourage use of services to contribute to improved population health outcomes. The approach created space for community participants to deliberate and surface a wide range of concerns with health services, collectively deciding on priority advocacy issues and approaches.

### Testing a community-level intervention with a randomized control trial

Phase 1 of ACT Health (2014–2016) included a series of five semi-annual CSO-facilitated dialogues between community members and health workers in 282 government health facilities; the use of citizen report cards to share information about health facilities with community-level stakeholders; and development of action plans, reviewed in follow-up meetings every six months. Phase 1 was evaluated through randomized control trial (RCT) research, which tested the impact of the citizen report cards (information) and community-level dialogues. When the RCT intervention began, CSO staff visited each intervention area selected in the randomization process, using standard tools to explain the intervention to health workers, community health workers, and local officials (elected and appointed). CSO staff provided mobilization lists and requested community health workers and elected officials to invite a variety of participants to attend dialogues.

In the initial dialogues, community-level participants developed action plans which specified issues of priority concern, actions to address those issues, and timelines for designated persons to take action. According to program records, across the 282 intervention communities, over 22,000 community members and 1,100 health facility staff attended the initial dialogues. During follow-up dialogues every 6 months, CSO facilitators guided participants through a review of progress against the action plans and documented reported updates. The combination of information, facilitated dialogues, and action plans was theorized to activate accountability and trigger health service improvements. The ACT Health RCT was modeled on the Power to the People study, which reported significant reductions in child mortality as a result of this relatively simple approach [6]. However, published findings about the ACT Health RCT indicated that in contrast to the Power to the People study, the community-level approach tested by the RCT had little impact on health outcomes [7, 8]. For the author’s analysis of the possible limitations of the RCT, see Bailey and Mujune [1].

Because the ACT Health RCT was designed to replicate an earlier study, this purpose determined the intervention and RCT study design. The RCT design intended to study the effectiveness of direct household (community-level) and health facility staff efforts on a set of five main outcomes and seven intermediate outcomes defined by external researchers. The RCT implementation excluded district-level officials from participating in dialogues due to researchers’ concerns that district officials’ behaviors towards health facilities randomly assigned to different treatment arms may be inconsistent. Likewise, facilitation guidelines developed to ensure consistency of implementation across the 282 facilities treated in the RCT encouraged community-level dialogue participants to focus on low/no cost actions that could be implemented locally. Additionally, the RCT design explicitly prohibited CSO staff from contacting communities or health workers in the months between facilitated dialogues as part of the RCT intervention. This limited CSO staff ability to observe and understand what happened between formal dialogues while the RCT was ongoing.

### Learning from a broader approach to citizen-led accountability

In its second phase, the ACT Health program expanded its activities, supporting multi-level advocacy campaigns that placed citizens in agenda-setting and action roles. While Phase 1 RCT implementation was on-going, the ACT Health consortium began planning for Phase 2 and developed this working definition of people-centered advocacy in 2015: “*People-centered advocacy is a systematic process owned and led by those affected by an issue using evidence to influence people with power at different levels to make sustainable change in practices, policies, laws, programs, services, social norms and values for the betterment of those affected by the issues*” [1]. Planning and preparation for Phase 2 took several months. Phase 2 implementation was approximately 18 months, from 2016 to 2018. In Phase 2, the ACT Health program supported 396 community advocates from 98 health facility catchments in 18 districts to directly monitor government health facilities; compile and analyze their own detailed data collected from multiple facilities; and ultimately develop and deliver their own advocacy asks and petitions to power-holders. This section provides an overview of key elements in the implementation of the people-centered advocacy approach.

#### In phase 2, ACT Health supported

After relatively light-touch facilitation and action planning during dialogues in Phase 1, during Phase 2 CSO staff facilitated iterative cycles of multi-day workshops, providing citizen advocates with space to diagnose root causes of problems, prepare to collect data, compile and analyze data, devise “asks” and develop advocacy strategies and target mandated powerholders. Specific activities in Phase 2 included: 1) ongoing horizontal organizing of community advocates from four to six health facility catchments who worked collaboratively to engage district officials for joint district campaigns; 2) a process of listing and comparing issues unresolved during Phase 1 dialogues in different communities, preference ranking/voting to select the shared priority advocacy agendas for Phase 2 campaigns; 3) coordinated, systematic independent community monitoring of government services across multiple locations followed by joint analysis of that data by all community advocates; 4) community-driven political economy analysis to identify key advocacy allies and target audiences for subnational advocacy campaigns; and 5) community advocates’ direct engagement of government actors at multiple levels through iterative advocacy campaign cycles.

#### Health facility selection for phase 2

Due to budget and capacity constraints, it was not possible to implement people-centered advocacy work which required more intensive, on-going support in all the 282 RCT health facility catchments from Phase 1. The implementing consortium selected 98 health centers from the 282 that had activities tested via the RCT. Each of the 18 districts had multiple health facilities selected for Phase 2. The selection of 98 facilities was guided by a few factors: 1) selection of facilities that had higher rates of unresolved issues during the community dialogues in Phase 1 (2014–2015); and 2) CSO facilitators provided insights on facilities that remained in need of more support to solve some issues from Phase 1.

#### Identification of community advocates

The approach in Phase 2 built on the relationships and activities in Phase 1. In the last facilitated community-level dialogues under the RCT, CSO facilitators introduced the people-centered advocacy approach, and explained the next stage of the work would be to select community advocates to organize and take some unresolved concerns from their communities up to higher officials. CSO facilitators supported participants to define the qualities and characteristics of community advocates. Selection criteria were participant-driven, and varied based on community expressed priorities. Some common shared selection criteria included: good relationships with community members, honesty, trustworthiness, ability to read/write, and the ability and willingness to speak up. Dialogue participants used their selection criteria to choose 396 community advocates (39% female, 58% youth under 35 years) who then organized within districts to collaborate on advocacy campaigns for the rest of Phase 2 activities. The program collected very limited demographic data on advocates. Some advocates had backgrounds as civil servants (teachers), others had no prior association with government or even paid work beyond farming. There was a lot of variety of backgrounds, but what unified advocates was willingness to volunteer and the selection by other community members.

#### Citizen-led issue prioritization

The database of issues/actions progress generated during Phase 1 community-level interventions was one input to the advocacy agenda-setting process, in addition to the problem analysis which included broader reflection and debate amongst advocates from multiple health facility catchments. During planning meetings, community advocates identified issues that they could not resolve at community level in dialogues with health facility staff. Some boundary criteria for the selection of issues for district advocacy were: 1) an issue had to be affecting all the health facilities represented by the advocates; 2) there had to be proof that this problem / issue could not be resolved after engaging directly with Health Center staff; 3) additional evidence collected about the issue through monitoring by community advocates had to demonstrate that the magnitude of the issue was high and the negative effects had to be confirmed through information validation by community member stories and reports; and 4) the issue had to be within the mandate of the district to address. In 14 districts advocates chose to work on health worker absenteeism, including late arrival and early departure compared to government guidelines. In two districts, advocates focused their advocacy on low staffing levels in facilities. In one district, advocates focused on improving lighting and infrastructure. In one district, advocates focused on environmental degradation, which they linked to health conditions in their district.

#### People-centered advocacy campaigns

Depending on the advocacy issue prioritized in each district, advocates’ subnational campaigns targeted elected political leaders, political appointees, and appointed technocrats serving in various capacities at village, parish, subcounty, and district levels. In 14 of 18 implementation districts, networks of community advocates focused advocacy campaigns on the complex, power-laden issue of health worker absenteeism. Once the advocacy campaigns began, community feedback meetings held by community advocates to provide feedback to the other community members about the progress of the advocacy campaign were intended to sustain the spirit of community dialogue, but the CSO-facilitated dialogues which were the focus of the RCT intervention in Phase 1 did not continue. With support from CSOs, advocates even directly engaged line ministries (health, finance, and local government), Parliamentarians, and the Inspectorate General of Government in Kampala with demands to close the many administrative loopholes that enable health worker absenteeism. The national campaign engagements differed from conventional CSO-led advocacy efforts because they were grounded in extensive prior work in districts. For multiple detailed examples of how the actions and tactics of community advocates evolved through iterative advocacy campaign cycles across the 18 districts and at national level, see Bailey and Mujune [1].

#### CSO facilitators in a support role

Following the principle of people-centeredness, CSOs took a back seat, finding ways to support community advocates to directly engage district and national-level officials on the issues that mattered most to them and their communities. After the initial program activities in each district (issue selection, data collection, data analysis, campaign planning), the CSO staff support roles in the second phase were guided by support plans derived in response to the needs of the community advocates during the evolving advocacy processes. During Phase 2 advocacy campaigns CSO staff maintained regular contact with advocates in training workshops, review meetings, phone calls, visits, coaching, and mentoring. The nature of this accompaniment support helped community advocates analyze the root causes of their priority advocacy issues and target asks to powerholders. CSO staff fostered role playing/simulating situations in the advocacy planning process to help provide a practical orientation for community advocates to prepare for navigating pushback from powerholders with confidence. Sustained CSO accompaniment of advocates through the 18 months of campaigns fostered a culture of “learning-by-doing” in ways not possible during the relatively bounded community-level intervention studied in the RCT. Through training, mentorship and accompaniment, the ACT Health program supported community advocates to understand and use government policies, processes, and mandates to build and deliver effective advocacy campaigns.

## Approach and methods

The ACT Health program implemented at this scale was made possible because of the initial interest in the RCT replication of the influential Power to the People study several years earlier. The external researchers (principal investigators) focused exclusively on the RCT on the Phase 1 activities. However, from the initial design stage, the implementing consortium and the funder had a broader interest in implementing (and investing in) an overall strategy that included but was not limited to the intervention designed for the RCT study.

After the RCT ended data collection, the relaxation of constraints from the research (such as the limited contact with intervention communities between formal induced activities) enabled more iterative strategic practice and more continuous program monitoring. While there was no planned or budgeted formal evaluation or assessment of the Phase 2 approach (or the entirety of the ACT Health approach), the authors’ saw an opportunity to leverage significant program monitoring documentation of the advocacy cycles in Phase 2. This combination of practice and monitoring makes the analysis of Phase 2 particularly valuable to learn from. The analysis on which this article is based explored in detail the intensity, scale and iterative nature of the people-centered processes involved in ACT Health’s second phase [1].

This paper is based on a systematic ex poste, inductive, practitioner-led analysis of the process monitoring data to understand the dynamics embedded in the cycles of citizen actions and government responses. Methodologically, this article demonstrates the value of analyzing process monitoring and program data to understand nuanced outcomes from direct engagement between citizens and the state to improve health services. Practitioner perspectives also open the ‘black box’ of implementation of community-led accountability programs.

### Data sources

The article draws on a range of evidence about Phase 2 of the ACT Health program, including program monitoring data, purposively gathered primary data from interviews and focus group discussions, follow-up information from program participants, and evidence of the program’s impact from news coverage and social media sources.

During Phase 2, each district-level advocacy campaign had a monitoring plan – specifying what changes the advocates expected as a result of their engagements. The community advocates led all campaign engagements, using report formats such as commitment logs to document contacts with government officials and record power-holders’ reactions, commitments and actions. The data from commitment logs and other reports generated by community advocates fed into joint reflection and revision of advocacy strategies during community advocates’ regular (approximately monthly) campaign review meetings. The data generated by community advocates fed into regular joint reflection sessions wherein advocates connected to review progress and revise their advocacy strategies. CSO staff also kept their own records and documented their trainings, review meetings, and interactions with community advocates. All these primary documents fed into a “Heat Map”, summarizing key actions of community advocates and reactions of government officials across all 18 districts.

### Analysis and triangulation

#### Review of the heat map monitoring data

The ACT Health advocacy Heat Map was an internal monitoring and external reporting tool updated by GOAL at three points in time (December 2017, May 2018, and December 2018). The authors’ analysis began with in-depth reviews of the 18 summary case descriptions and ratings of government responses in the December 2018 Heat Map. The authors reviewed each district campaign description, revisiting source documents or seeking clarification from involved parties to add additional details to the Heat Map. To capture campaign developments after formal project support ended, the authors drew on updates from community advocates, often in the form of WhatsApp messages, text messages, or phone calls.

Through the iterative systematic review of the Heat Map case descriptions, the authors refined the original criteria for classification of government responsiveness: “red” (officials largely unresponsive), “yellow” (officials made commitments but implementation was limited), and “green” (officials implemented commitments). This review revealed some cases where subnational government actors actually implemented actions beyond the “asks” of community advocates, leading the authors to add a fourth category of responsiveness—“purple” (officials implemented actions beyond campaign commitments). During the data validation, the authors applied these definitions very strictly, revisiting prior program staff ratings of the responsiveness of officials in each district. A conservative application of rating criteria led the authors to downgrade prior assigned ratings of the responsiveness of eight districts from green to yellow. The authors also upgraded the rating of three districts from “green” to the new “purple” category.

This summary case description extracted from the more detailed “Heat Map” gives an example of the data analyzed and illustrates a case of a district with the highest level of responsiveness (rated purple): *“Community advocates contributed to Agago District (rated purple on the heat map) removing 13 “ghost workers” (names of people still on the register who had left the district, died, or retired) from facilities the advocates were monitoring, and hiring 6 additional health workers for 2018. Advocates went on to petition the Chief Administrative Officer (a centrally appointed technical official), and the Local Council V Chairperson (the highest elected official in the district), to request that they hire more workers beyond the replacement of ghost employees. In April 2018 the Local Council V Chairperson committed to support advocates to present their case to the District Council’s Health Committee. Community advocates continued to follow up on issues of unexplained staff absence, staff transfers without replacements, and granting of leave without due consideration of gaps, in three problematic facilities. Based on a request from the Resident District with effect from November 2018. The Agago Resident District Commissioner provided training to advocates to use the district’s teacher monitoring tools.”*

#### Triangulating against source documents

During on-going monitoring, the program also collected a range of evidence such as photographs, newspaper articles, and letters from government officials documenting events and developments during the program. The authors leveraged such source documents to triangulate against the Heat Map case descriptions and validate observed outcomes.

#### Focus groups and interviews

In June 2018, the authors visited three implementation districts and conducted three focus group discussions with a total of 15 community advocates and five interviews with government officials. The three districts were not representatively sampled for the 18 intervention districts, but included one rated “yellow” (commitments made), one rated “green” (commitments implemented), and one rated “purple” (commitments beyond campaigns). The corresponding author did one additional interview with three civil society organization colleagues from two of the four organizations in the implementing consortium. Overall, the role of these focus groups and interviews was to allow one author who was not in Uganda during the implementation of the advocacy campaigns to have the opportunity to directly interact with and hear the lived experiences of advocates and officials they engaged. These interviews surfaced participant-reported insights into change processes, and the reports from respondents have been critically interpreted as part of the overall analysis conducted but are not the sole source of data for any analysis or finding. The information gathered in these engagements with program participants was useful in helping frame the iterative review and systematic analysis of Heat Map data.

### Limits of this study

A key challenge to the analysis of the advocacy campaigns was that the accompaniment and support offered to people-centered advocacy campaigns was an iterative and learning process, thus less amenable to standardized implementation and monitoring tools than the approach in Phase 1 which was governed by the RCT and therefore required strict adherence to implementation protocols. The highly relational nature of advocacy means tracing its direct causal impact is always challenging [9]. The data sources and methods grounding this systematic analysis did not use any standard qualitative comparative research methodology and lacks the ‘gold standard’ rigor of experimental research.

This article is shaped by the positionality of the authors’ and the respective roles in the design and implementation of the ACT Health program over several years. The early program design was informed by existing knowledge (tacit and explicit) on bottom-up health accountability work, particularly in the Uganda governance context. Learning-by-doing informed the evolution of Phase 2 implementation. The authors have attempted to compensate for bias through triangulation of data and critical application of responsiveness ranking criteria. As reflective practitioners committed to learning about the potential and pitfalls of participatory governance, the authors’ firsthand knowledge of the ACT Health program enables critical exploration of varying outcomes of a multi-level strategy in practice. These insights shine light into the black box of implementation.

## Results

This section of the article focuses on findings suggested by systematic review from the program’s own approach to assessing the responsiveness of government actors to the citizen-led advocacy campaigns—a “Heat Map” developed to provide a grounded account of the changes in response to the 18 community-led advocacy campaigns supported by the ACT Health program.

Systematic analysis of this rich body of data from the 18 subnational campaigns revealed patterns of government responsiveness to citizen-led advocacy. It provided insights into the extent and nature of the work needed to facilitate and support such advocacy, the form and degree of responsiveness by government officials at different levels of the system, and of the kinds of changes in health service provision this engagement brought about. A review of the literature indicates that few such approaches have been analyzed at scale or with such close attention to the multiple and iterative nature of the inputs, and the non-linear and varied responses and outcomes. This article aims to show that change from below is possible – rarely simple and never guaranteed – but also that rigorous understanding of those changes is also possible.

**Fig. 1:**
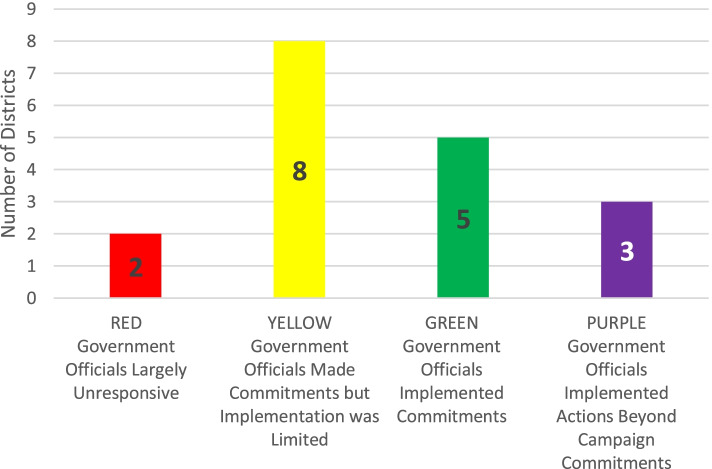
Subnational Government Responsiveness to Community Advocates’ Campaigns (as of June 30, 2019)

A cautious and conservative rating of the responsiveness of officials suggests that in eight of 18 districts officials either fulfilled or surpassed the commitments they made to community advocates. The data analysis suggests two unresponsive districts (red), eight districts where officials made commitments (yellow), five districts where officials implemented commitments (green), and three purple districts so rated because officials took actions beyond the advocates’ campaign asks (Fig. 1). This results section categorizes and describes a range of government responses suggested by the analysis and ends with a description of some suggested outcomes for participating community advocates.

During campaign review meetings, many advocates reported that their ongoing monitoring in facilities did not show significant improvements in attendance of health workers to the advocates’ satisfaction, even when advocates reported officials’ responsiveness to their campaign asks. This is unsurprising, given the complex nature of absenteeism [10]. The more nuanced outcomes suggested in the analysis emerged from the cycles of interaction and direct engagement between community advocates and progressively higher levels of government actors. While some of these responses are to be expected or at least hoped for when citizens voice their needs with government health officials, many of the types of responses elicited were unexpected or appear to be identified only rarely in health accountability programming.

### Government responses

#### Increased government monitoring

The findings suggest that community advocates’ bottom-up monitoring and advocacy triggered increased government monitoring by district officials. In Uganda, officials commonly complain that they lack the resources to make regular support-supervision visits to health facilities, but independent monitoring by citizens appears to have exposed gaps in the management tools on which district officials rely. In 13 of 14 districts focusing on absenteeism, the evidence indicates that district-level officials went to verify the absenteeism data (evidence) presented by community advocates. As one district official noted:

*“As duty bearers, we have an oversight role, but we are limited and cannot always be there. We entrust those posted to manage their work. The biggest problem is ‘organized absenteeism’ where health workers make their own informal timetable. Community advocates helped us discover this practice. We have taken a serious intervention.”* [11]

#### More downward accountability and proactive transparency

Documented government responses also included proactive transparency by government officials, to and beyond advocating communities. The Resident District Commissioner of Manafwa District (rated green on Heat Map) went on the radio to share the outcomes of his monitoring visit to the health facilities. The Mubende District (rated yellow on Heat Map) Health Officer called health assemblies in all five facilities to share his findings from the data verification process, acknowledging the community advocates’ role in triggering the monitoring investigation during public debrief sessions. These examples illustrate closing of the feedback loops (reporting back to those who requested action), and seem to suggest a degree of answerability / downward accountability (reporting back to community advocates rather than only upwards to their own supervisors). At the health facility level, program monitoring found notices posted by health workers proactively disclosing the duration of their absence from duty with contacts of the person in-charge during their absence. The suggested micro-shifts in power dynamics are significant in the Uganda context, and while visible to practitioners, they are very difficult to capture in survey data.

#### Countering backlash

There were also important instances of government officials reportedly responding to backlash against community advocates by frontline workers or local level officials, in ways that may suggest slightly altered power dynamics of accountability. Challenging vested interests and the status quo can trigger negative reaction, backlash, and possibly retaliation. Because hidden power dynamics often shape citizens’ interactions with government employees, simulations and role-plays during training sessions and workshops helped prepare community advocates for possible pushback. When community advocates began monitoring facilities, some health workers resisted or retaliated. In one district (rated yellow on Heat Map) the In-Charges of two health facilities banned community advocates from accessing services, but advocates reported this to higher officials. In response, the Resident District Commissioner (a political appointee) put the In-Charges “on notice” that no one would be refused treatment for exercising their rights as community members and citizens. The findings here seem to suggest the potential of community advocates to leverage new relationships with higher-level officials to protect themselves against further retaliation by local service providers—representing a possible shift in micro-power dynamics between community members and health workers.

#### Application of sanctions

In several cases, the data suggests that advocacy also encouraged some government officials to enforce sanctions. In eight districts, officials reportedly summoned health workers implicated by community advocates to issue verbal warnings or instructions. In six districts, officials issued warning letters to errant staff. In Omoro District (rated green on Heat Map), officials planned to withdraw salaries for all health facility staff confirmed absent from duty for more than 15 days. Two districts reinstated or reinvigorated Rewards and Sanctions Committees to take up disciplinary actions. However, the disciplinary mandate for government employees at the subnational level lies with the District Service Commission, which was not active in any district. This illustrates the complexities of Uganda’s local governance structures, and under-resourced mandates to enforce accountability.

#### Bolstered budget allocations

Community advocates actively used government-invited spaces, including subcounty and district annual budget conferences and district council meetings where citizens can, in theory, directly access leaders. The analysis suggests that some advocacy campaigns seem to have succeeded in influencing budgetary allocations for particular health services. In one district (rated green on the heat map), subcounty officials allocated funds for electricity and lighting in six of seven health centers included in advocates’ petitions. In Bukedea District advocates’ identified lack of staff housing as a root cause of health worker absenteeism and requested budget allocation for this. Findings suggest that in response, Bukedea District (rated purple on the heat map) allocated district funds to three facilities and officials provided the technical specifications to advocates and provided them guidance on monitoring the construction. Direct, causal impact claims linking any single, discrete budget (or other) advocacy effort to a resource allocation are not straightforward and thus not an explicit claim of this analysis; this is not a new challenge in assessing advocacy impacts [9]. Although community advocates’ work was unlikely the sole impetus behind observed budget allocations, these examples may suggest the power of informed citizen advocates and their campaigns.

#### Official government recognition of advocates

A final set of responses was official recognition by government, which appears to bring both advantages and potential challenges for community advocates. Government officials in 6 of the 18 districts provided letters introducing advocates as community volunteers. Advocates reported that they could present such letters to health workers or other lower-level officials, in case community advocates had difficulty accessing facilities for ongoing monitoring. Formal recognition of this kind is highly valued by citizens in the Ugandan context. However, this recognition may imply the transfer of monitoring responsibilities from mandated government officials to community advocates. Practice-based reflection from Mozambique highlights that the transfer of responsibilities from government to citizens potentially inverts roles and can further minimize the state’s performance of its mandated duties [12]. Other scholars have suggested that such recognition and shared responsibility can be considered a form of co-production, whereby citizens and the state share responsibility for monitoring in resource-scarce settings [13, 14]. In the Uganda context, findings seem to suggest that higher level officials see advocates as their ‘eyes and ears’ in communities and one possible negative outcome of this may be to co-opt or exploit advocates’ monitoring efforts in health facilities. Likewise, advocates may seek a closer affiliation with the state if they expect it may lead to opportunities for remuneration, formal employment, or other perceived benefits. These dilemmas illustrate both the power and the complexity of independent citizen-led monitoring and accountability efforts.

#### National policy-makers also responded to community advocates

Organized national-level engagement culminated in April 2018, when ACT Health organized a symposium for community advocates from 14 districts and national stakeholders to discuss community monitoring of primary health care. With CSOs to help map policy, understand opaque power dynamics, and ‘open doors’, community advocates directly engaged with the Speaker of Parliament, Inspectorate of Government, three key line ministries (Health, Public Service, and Local Government), and the Office of the Prime Minister. The orchestrated campaign engagements at the national level required significant civil society support, but program data revealed that advocates from two districts directly engaged national-level authorities too, escalating issues not addressed by their district-level officials. As the community advocates worked their campaigns, they expanded their understanding of government accountability mechanisms—including the office of the Inspectorate of Government (IGG). The case of Mubende District (rated yellow on the heat map), illustrates the direct engagement by advocates with this national oversight body. By June 2018, the Chief Administrative Officer (a central-government appointee) had a good understanding of petitions previously submitted by the advocates, but no district official had taken concrete action. In August 2018 advocates directly petitioned the central regional office of the IGG, which conducted its own investigation into health worker absenteeism in December 2018. In response to the IGG report, district officials took immediate action on the unqualified and/or absent staff mentioned in the community advocates’ petition. This escalation of concerns in response to subnational administrative inertia may suggest that the persistence and confidence of advocates to reach more senior government actors can trigger top-down action. The findings seem to suggest that citizens’ ability to directly trigger the horizontal oversight mechanisms of the Inspectorate of Government, is an important tactical approach to claiming accountability [15, 16].

### Outcomes for participating community advocates

At subnational levels, while their efforts were uneven, the findings suggest that most community advocates worked collectively to advance district campaigns. The joint agenda-setting process, coordinated monitoring of multiple facilities, and sustained joint advocacy campaigns (over approximately 18 months) were accomplishments in their own right. The analysis of the ACT Health program may suggest signs of deepening democratic citizenship among community advocates even in this donor-funded intervention.

The analysis of evidence suggests that many community advocates developed or enhanced their reputations as leaders, taking on additional advocacy issues. Interviews with advocates from three districts in June 2018 all surfaced examples of community members contributing resources (cash and in-kind) to campaigns, mobilizing for joint advocacy actions, or reporting problems to advocates [1]. It is important to understand the success of community advocates’ local resource mobilization in the Ugandan context, in which induced participation in projectized approaches render more organic coalition-building and organizing very challenging. The evidence suggests that advocates in almost half of the 98 health facility catchments used their skills and knowledge to take on additional advocacy agendas. The special campaigns initiated by advocates seem to demonstrate use of knowledge and skills to expand the scope of their advocacy work.

For many of the 396 community advocates, testimonies and reports seem to indicate that working on advocacy campaigns was transformative for several participants. On reaching district officials, one advocate said: *“We have engaged the CAO [Chief Administrative Officer], RDC [Resident District Commissioner], DSC [District Service Commission] and because of these achievements, I’m so confident I can even speak to the president about ACT Health people-centered advocacy – I’m very comfortable.”* [17] For this advocate, the testimony suggests that engagements were empowering, regardless of any further outcome. Another community advocate described an educational journey: *“The dialogues were nursery school. What we have done up to now with advocacy is primary school. Now, I want to go to secondary school.”* As a follow-up question, researchers asked advocates to assess the national level campaign, which another advocate responded: *“now, that one is university!”* [17]*.*

During an off-cycle nation-wide special local council election in 2018, 47 community advocates (over 10%) were elected as village Local Councilors (LCIs) [1]. While it is possible that the people interested in becoming community advocates would have sought elected office anyway, the analysis of findings may suggest that people-centered advocacy processes fostered an understanding of government policy and practice that may have enhanced their public service capabilities. Building advocates’ civic knowledge of government policy and decision-making and expanding their skills to directly engage officials can foster the emergence of democratic citizenship, a suggestive finding echoed in other work [18]. While this may not translate to improved health outcomes in the short term, political learning, capabilities, and the exercise of citizenship are important for more transformational change [3, 19–23]. In the Ugandan context, Namisi has described raising “civic competence” as “reawakening the sleeping giant” of a disempowered population [23].

## Discussion

Analyses of citizen-led health accountability interventions targeting multiple levels of governance are rare in the literature. Much of the intervention-based research on social accountability in health focuses primarily on provider–patient dynamics [24–26]. Research into accountability initiatives often assumes that the core mechanisms of change can be uncovered through singular and linear interventions, frequently centered on the provision of information about public services [27]. Experimental research is suited to studying relatively simple interventions—tools or “widgets” [28] and short causal chains amenable to standardization, rather than to testing broader change theories or dynamic social processes [29–33]. Much of the experimental evaluation literature has also focused on the role of NGOs in community-based monitoring, rather than on the roles of communities themselves [21].

Much of the conceptually-oriented literature on accountability points to weaknesses in the project-based technocratic approaches, emphasizing the need to focus on power shifting in development projects [26, 28, 29, 34–36]. Many scholars from multiple disciplines and methodological backgrounds place increasing emphasis on context-driven approaches, stressing the importance of seeing interventions or processes in the context of larger histories of citizen–state engagement [34, 36]. While vital, this call to focus on power-shifts in context takes significant work to implement in practice and study with formal research designs.

With a few notable exceptions, iterative cycles of action and response of the kind explored in this analysis of the ACT Health strategy are infrequently discussed in the formal literature on health accountability [22, 37]. Apart from some implementation science literature, little attention is paid in the formal literature to the nature, detail, and intensity of the inputs and processes through which citizens seek accountability, and in particular to the interpersonal, relational, and trust-based nature of facilitation and support for citizen-led advocacy on health services. The analysis of 18 campaigns documented in the “Heat Map” partly responds to the noted challenges of capturing contextual variation across geographic program areas, as this often requires deeper ethnographic sensibility and highly adept, iterative monitoring—both of which are challenging to implement at a scale as large as the ACT Health program analysis [20].

Overall, despite the limits of the study noted under “Approach and Methods,” this practitioner-led analysis surfaced important context-aware insights into the possible mechanisms through which observed outcomes emerged. This grounded account adds to the body of explicit knowledge on multi-level, context-driven, people-centered accountability work in practice. The remainder of this discussion section uplifts these insights.

### Accountability research often focuses on discrete, measurable interventions; but multi-level approaches (like the ACT Health strategy in phase 2) tend to be less studied

The intervention tested in the ACT Health Phase 1 RCT was largely limited to community-level participants (community members and health workers). In all 18 districts, analysis seems to suggest evidence of direct, sustained, citizen-led monitoring and engagement with elected and appointed officials at the village, subcounty, and district levels. Despite many challenges, in many districts, the findings do suggest community advocates coordinated and applied pressure on target audiences through multiple cycles of engagement. Evidence of citizens’ direct and sustained engagement with government officials across multiple levels is an achievement in and of itself in this context. In one focus group discussion, advocates themselves seemed to distinguish between the process and outcomes in Phase 1 community-level dialogues versus the subsequent advocacy campaigns in Phase 2:

*“There is a very big difference. With dialogues, we would stop at the sub-county – we were not known at higher levels. Now with PCA [people-centered advocacy] we open different offices at higher levels. RDC [Resident District Commissioner] will recognize we are from a specific boma [neighborhood]. Now at the health center the lowest member gets treated fast and better and that was not happening during dialogues.”* [38]

While the literature suggests that it is especially difficult to pursue approaches that transcend the local level in weak or oppressive states [18], the ACT Health program seems to indicate that even in contexts dominated by complex political, administrative, and power hierarchies it is possible to create spaces for citizen–state engagement across multiple levels. The overall findings may suggest that citizen-led engagement engendered a wide range and degree of responses, with government officials in eight districts meeting or exceeding their commitments to action.

### Building a bottom-up campaign to engage national-level officials requires time and technical support

The national campaign engagements differed from conventional CSO-led advocacy efforts because they were grounded in extensive prior work in districts. Building from district campaigns, the findings suggest that ACT Health consortium at least helped community advocates directly engage with multiple audiences at the national level. The time invested in planning and the bottom-up approach to agenda-setting and multi-level advocacy meant that coordinated, visible national-level campaign actions began in the last six months of the project. Given the short timeframe for the coordinated national campaign on absenteeism, and the limited follow-up at the national level after the end of the formal funded intervention, no real changes resulted from pledges by national-level duty-bearers. Still, the work yields lessons for future practice. While the process of intra-district organizing and executing 18 subnational campaigns was viable without material incentives to advocates, reaching the national level was more challenging. Many community advocates had never been to Kampala, let alone to Parliament. The findings indicate that significant technical support, accompaniment, and time were needed to consolidate data, research policy, map power holders, target campaign ‘asks,’ and execute national-level engagements. The evidence may suggest that collective national-level engagements may require more resources than citizen groups can raise independently, yet the ACT Health program evidence does suggest that national-level actors (for example the Inspectorate of Government) are potentially within direct reach of coordinated district-wide campaigns. It is difficult, but the findings from the ACT Health suggest that it is possible to open the doors of national government officials to citizens.

### Community advocates can work to activate subnational governmental checks and balances

District level campaigns had to navigate three parallel elected and appointed governance structures: the Chief Administrative Officer (a centrally appointed bureaucrat), the Local Council V Chairperson (elected), and the Resident District Commissioner (executive branch appointee). To activate checks and balances at the subnational level, the findings suggest that community advocates engaged leaders in all positions—often approaching one leader with requests to influence or pressure another duty bearer to act. In ACT Health, districts with top leaders from the same political party were more likely to deliver on commitments/pledges to advocates. For example, in one district, rated yellow on the Heat Map (commitments made with limited implementation), the Local Councilor (elected) was a prominent opposition leader who embraced the advocates and committed to engage other duty-bearers on absenteeism. The Resident District Commissioner (a presidential appointee and member of the ruling party) feared that community advocates were working against his party and disregarded the issue tabled by advocates. Patterns in the ACT Health implementation districts seem similar to findings of recent comparative work in Uganda suggesting that collaborative coalitions at district level—among politicians, bureaucrats, health sector professionals, and CSOs—“with the capacity and commitment to devise and enforce innovative approaches to governing the sector” drive good service delivery [39]. It may be the case that public servants who already embrace the value of citizen involvement will be more responsive, but this was not a hypothesis tested in this study of the ACT Health program. The role of political party affiliation, competition, and coalitions of public servants in shaping government responsiveness to citizens are areas for future studies.

### People-centered advocacy campaigns can trigger synergy between bottom-up and top-down accountability efforts

The Power to the People and ACT Health RCTs both suggested that synergy between bottom-up and top-down approaches to accountability could be productive, yet neither RCT was designed to trigger or study those dynamics [6–8]. The broader ACT Health strategy anticipated that citizen engagement with higher-level officials would be necessary to see more systemic responsiveness and accountability to citizens, and Phase 2 encouraged synergy between bottom-up and top-down approaches. A central strategy of most advocates was to activate ‘top-down’ official oversight and downward accountability from government actors to citizens, which is critical for triggering responsiveness and more transformational changes. Some higher-level officials were initially skeptical or resistant, but the evidence suggests that many came to appreciate the earnest independence and the detailed monitoring work of advocates. In 13 of 14 districts, officials did their own independent monitoring to verify reports of health worker absenteeism—a finding that may suggest that advocates effectively triggered top-down oversight. In Mubende, advocates were unsatisfied by limited responses from district officials and triggered an independent investigation by the Inspectorate of Government (top-down oversight from national to subnational officials). Community advocates in Bundibugyo threatened to appeal to the Inspectorate of Government, and that threat alone appears to have triggered district-level action [1]. These findings seem to suggest the importance of a multi-level approach to demanding accountability.

### Strong process monitoring and analysis grounded in reflective practice can surface subtle shifts in power dynamics

When community advocates began monitoring facilities, it was reported that some health workers resisted and, in a few extreme cases, retaliated (see backlash discussion above). Upon learning that advocates had reached district officials, the evidence suggests that some Health Center In-Charges called community advocates in to negotiate with them after having treated them badly in prior interactions [1]. Examples of proactive transparency at health facilities (e.g., posting of staff names and duty rosters) and district-level officials reporting findings back to advocates after their rounds of top-down monitoring (completing feedback loops) may suggest promising signs of increasing downward accountability (or at least answerability) to citizens. Feedback from interviews with community advocates informs the understanding of possible drivers of suggested responsiveness:

*“Community used to take the health workers as the president. Now, health workers know that the community knows their rights and responsibilities. The relationship has changed greatly*.” [38]

*“At start, people like the LCI [village elected local councilor], other community members and health centre staff couldn’t believe we could go as far as the district government. They thought at most we would stop at the sub-county and now they hear we have gone to the RDC [Resident District Commissioner]. The RDC invited us to attend a meeting with state house [president’s office] and the LCV [district elected local councilor] started to respect us more after that.”* [38]

These examples suggest some advocates’ experienced changes in power and accountability dynamics. In the Uganda context, these subtle changes are significant, and may encourage citizens’ continued engagement with the state. Survey-based research methods and quantitative analysis by external researchers far-removed from implementation context may fail to capture these more subtle signs of government responsiveness, and the complex relational outcomes (such as signs of shifting power dynamics) many hope to see from citizen-led accountability efforts.

## Conclusions

Analysis of efforts to strengthen accountability infrequently address the non-linear, geographically uneven realities of supporting citizens to hold health system service-providers and power-holders to account. This article shared findings from a practitioner-led study of the changes brought about by one multi-level program for strengthening accountability for health in Uganda. The patterns and meanings identified are valuable because some of the most significant changes for community advocates are the hardest to measure—especially for outsiders relying on survey data alone. Close-grained analysis of the ACT Health program found that enabling community advocates to identify and campaign for the health services they need at multiple levels made important changes to the dynamics of health service provision. The analysis of the 18 district campaigns surfaced examples of increased health system accountability to citizens, even under challenging social, political, and administrative conditions found in Uganda.

Experimental research is often not amenable to the more flexible and reflective accountability work that many in the field increasingly see as having more potential to improve governance or health outcomes. Robust process monitoring data consolidated into a Heat Map combined with practitioner’s analytical insights made visible district level variation in government responsiveness. What makes this methodological exploration innovative is the mindful, action-oriented monitoring across multiple levels in 18 different districts. The compilation of Heat Map process monitoring data is a more ethnographically inspired approach to document the range and degrees of responsiveness, the analysis of which is crucial for understanding why something happens or does not. This is a contrast to more conventional research data focused on aggregating quantitative data and determining average treatment effects. Investment in strong process monitoring and analysis leveraging practitioners’ tacit knowledge can surface negative and positive outcomes—all of which are essential for understanding and contextualizing changes from accountability processes.

As promising as the cycles of citizen action and government responses evident from the people-centered advocacy approach are, critical practitioners are not naïve about the limits and risks of induced interventions such as the ACT Health program. In contexts of constricting civic space, independent monitoring by citizens alone risks placing excessive burdens on those closest to the problems, with the least resources and power to directly solve them. Civil society organizations (CSOs) can maximize efforts to put citizens most directly affected by problems in the agenda-setting and direct advocacy roles. CSOs must also prepare citizens to mitigate backlash, and CSOs must actively monitor and intervene if appropriate. The right strategic support from funders and CSOs can create an enabling environment for horizontal organizing and collective voice, increasing the power of community members vis-à-vis government officials. Long-term, iterative, and people-centered approaches targeting multiple levels of governance may contribute to creating conditions for deepening democracy and positive change over time.

## Data Availability

The datasets used and/or analysed during the current study are available from the corresponding author on reasonable request.

## Abbreviations

ACT: Accountability Can Transform
RCT: Randomized control trial
CSO: Civil society organization
